# Newborn Screening for Primary Immunodeficiencies: The Gaps, Challenges, and Outlook for Developing Countries

**DOI:** 10.3389/fimmu.2019.02987

**Published:** 2020-01-30

**Authors:** Zeinab A. El-Sayed, Nesrine Radwan

**Affiliations:** Pediatric Allergy and Immunology Unit, Children's Hospital, Ain Shams University, Cairo, Egypt

**Keywords:** newborn screening, primary immunodeficiency diseases, TRECs, KRECs, MENA region

## Abstract

Primary immunodeficiency diseases (PIDs) are genetically inherited diseases characterized by an increased susceptibility to infections, autoimmunity, lymphoproliferation, and malignancies. PIDs are under-diagnosed and the registered cases and reported prevalence are far below the estimated numbers especially in countries with large population and high consanguinity rates. Delays in diagnosis yield major morbidities and mortalities with resultant increased economic burden. Newborn screening using TRECs and KRECs, currently being implemented in some countries, is aimed through early diagnosis, to overcome the delays in the diagnosis and hence the poor outcome of some of the severe PIDs. However, the limited resources in developing countries challenges the implementation of newborn PID screening programs. There are considerable gaps in our knowledge that must be bridged. Setting the norms of TRECs and KRECs for each country is needed. Furthermore, some PIDs that might present in the neonatal period could not be detected by the current screening programs, and their diagnosis requires clinical expertise. Not to mention, local guidelines for the management of patients diagnosed by NBS should be set forth. Also, in the absence of NBS, clinicians should be aware of the early manifestations of PID. All these mandate conducting studies genuine to each country, developing programs for raising public awareness and clinical training of physicians to attain the required immunological skills.

## Introduction

Primary immunodeficiency diseases (PIDs) are genetically inherited diseases that are characterized by an increased susceptibility to infections, autoimmunity, lymphoproliferation disorders, and malignancies (1). It was previously considered rare; however, recent studies showed that almost 1% of the population will have a PID (2, 3). The number of cases globally diagnosed in 2018 is 94,024 with an increase of 21.8% than in 2013 (3). The prevalence of PID varies from one region to another, being higher in the USA, followed by Europe, Latin America, Middle East, Asia, and finally Africa (3). Currently, more than 320 genes have been discovered to cause PID with a wide range of clinical phenotype (4).

## PIDs: The Impact of Under and Delayed Diagnosis

PIDs are usually detected in early childhood, but, in some cases, the diagnosis can be delayed (5). This can be attributed to diagnosis lag. The diagnosis lag, the time elapsing from the initial presentation to establishing a diagnosis, was reported to be prolonged, even with the most advanced healthcare systems, surprisingly reaching 12.4 years in the USA (6). In a recent worldwide survey on X-linked agammaglobulinemia (XLA), there was a wide variation in diagnosis lag, with 34% of the participating centers reporting delays beyond 2 years (7). This may be attributed to the lack of awareness, the scarcity of specialized centers, the poor resources for diagnosis, and, finally, the wide range of clinical phenotype.

The delay in diagnosis is of considerable concern with respect to the consequences of repeated and severe infections such as bronchiectasis and the complications resulting from live attenuated vaccinations such as BCG and oral poliomyelitis vaccine (OPV). Disseminated BCG-osis has been a well-known complication with a reported incidence of 64% among patients presenting with disseminated BCG-osis (8). OPV can replicate for a prolonged period in PID patients, which can cause an increase in transmissibility and neurovirulence of the virus, which, besides carrying the hazard of vaccine associated poliomyelitis in PID patients, can theoretically be a potential threat to the community (9). These negatively impact the morbidity and mortality of PIDs and the outcome of therapies including hematopoietic stem cell transplantation (HSCT).

## Newborn Screening (NBS) for PIDs

There is a compelling need for having an early detection program, given that early detection of PID dramatically improves quality of life and life expectancy through prompt implementation of appropriate medical interventions and saves much of the expenses in medical care. Hence, neonatal screening was suggested for the purpose of early recognition of treatable, severe forms of PIDs with profoundly low T and B cell numbers (10, 11). This is done through quantifying T-cell receptor excision circles (TRECs) and kappa deleting-recombination excision circles (KRECs) (10).

TRECs are small circular DNA by-products produced during T cell receptor recombination in naïve T cells (12). They were found to be specific to naïve T cells and their levels decline with age in healthy individuals and HIV patients. TREC copy numbers are measured by quantitative reverse transcription polymerase chain reaction (qRT-PCR) (13). They are reduced or absent in severe combined immunodeficiency (SCID) and other T cell lymphopenias (12). The assay is highly sensitive for detection of SCID with severe T cell lymphopenia, albeit not for SCID variants. The assay allows the identification of some other PIDs such as complete DiGeorge syndrome, leaky SCID, and ataxia telangiectasia (14). However, there are some PID reported to have normal TRECs (see Table 1). In 2005, the TREC assay was applied for large-scale screening in the USA (16), and nowadays, it is applied in most states. This led to a higher than expected incidence of SCID reaching 1/58,000 (17).

**Table 1:** Causes of normal TRECs and KRECs.

**Normal TRECS**	**Normal KRECS**
Common variable immunodeficiency	IL2RA
Partial ADA SCID	JAK3
MHC Class II	CD40L
FOXP3	Selective IgA deficiency
CD40L	IL7RA
IL-10RA	

*ADA, adenosine deaminase; CD40LG, CD40 ligand; CVID, common variable immunodeficiency; IgAD, IgA deficiency; IL10RA, interleukin 10 receptor, alpha; IL2RA, interleukin 2 receptor, alpha; IL2RG, interleukin 2 receptor, gamma; IL7RA, interleukin 7 receptor, alpha; JAK3, Janus kinase 3; KRECs, k-deleting recombination excision circles; SCID, severe combined immunodeficiency; TRECs, T-cell receptor excision circle. Quoted and modified from Serana et al. (15)*.

KRECs are B cell products produced during rearrangement of the variable, diversity, and joining domains of the B cell immunoglobulin kappa gene (18). It was first developed for assessment of patients with antibody deficiency disorders and monitoring B cell recovery following HSCT (19). In 2011, the utility of the KREC assay in identifying XLA and XLA-like diseases in neonates was demonstrated (18). However, there are instances of normal KRECs level happening in some PIDs (see Table 1).

In spite of the added costs by measuring both TRECs and KRECs level, it allows identification of more types of PIDs such as late onset ADA deficiency and some cases of Nijmegen breakage syndrome (20). Also, measuring them is considered less expensive than flow cytometry assay, which is not only expensive, but needs a lot of training and not available in all countries (21). Adding to the merits of NBS, the cost reduction calculated for early diagnosis was $85,882 and $55,882 for patients treated with immunoglobulin replacement therapy (3). Finally, the survival rate of infants transplanted before 3 months and diagnosed by NBS was 94% in comparison to those transplanted later and had infections (50%) (11, 22).

**Figure 1:**
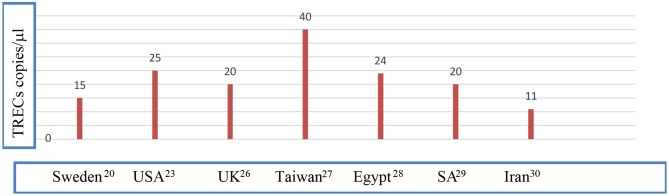
Different cutoff values of TRECs in different populations. Data were quoted from Borte et al. (20), Kwan et al. (23), Adams et al. (26) Chien et al. (27), Salem et al. (28), Al-Mousa et al. (29), and Nourizadeh et al. (30). NBS, newborn screening; SA, Saudi Arabia; UK, United Kingdom; USA, United States of America.

Caution is to be pursued while assessing the results of NBS. Preterm infants suffer from many problems, which yield the diagnosis using newborn screening challenging (10) and samples taken prior to the 32nd week of gestation to be repeated subsequently to exclude abnormal results due to physiological immaturity (23). Whether prematurity *per se* is a risk factor of having low TRECs or not is debatable (23). Multiple congenital anomalies and congenital heart disease are associated with low TREC levels (12). Maternal drug history is important since maternal intake of azathioprine was associated with low KREC level in offspring (24, 25). In addition, ethnic differences in cutoff levels of TRECs and KRECs exist and remain to be studied on a larger scale [(20, 23, 26–30); Figure 1].

The future is now heading toward protein-based screening methodologies to identify infants with complement and granulocyte disorders by measuring specific granulocytes proteins and some complement components using reverse-phase protein microarrays for determination of complement component C3 levels in DBS collected at birth. Targeted DNA sequencing has previously been employed for screening selected diseases, such as glutaric acidemia type I and cystic fibrosis and has also been described as a potential screening method in familial hemophagocytic lymphohistiocytosis (FHLH) due to mutations in *UNC13D* (13).

## MENA Region and the Challenges Ahead

The incidence of consanguineous unions is almost 65% in the MENA region^1^, which is significantly higher than in any other parts of the world (31). Consequently, the incidence of PID is expected to be 20 times greater in Middle Eastern countries compared with North America and Europe (32) as most PIDs are autosomal recessive diseases. However, the absence of national registries in most MENA region countries makes it difficult to determine the actual numbers. The estimated prevalence of PIDs in MENA region is 0.8–30.5/100,000, based on pilot studies and reports from different centers (32).

Based on the data collected from different publications (Egypt, Israel, Kuwait, Morocco, Saudi Arabia, Tunisia) (33–38), combined immunodeficiency (CID) ranks as the most common PID, whereas in Turkey (39), antibody deficiency is the most prevalent (32), and in the latest Iranian National registry too (40). The mean age of diagnosis is 2 years in the MENA region (32). A pilot study on NBS using TRECs and KRECs in Saudi Arabia showed an increase in the number of patients diagnosed to have SCID reaching an incidence of 1 in 2,906 live births (29), which is a much higher incidence than in California (1/65,000) live birth (41). This highlights the importance of implementing NBS for the early detection of SCID in MENA region countries. Currently, few countries of the MENA region have taken to newborn screening. These are Qatar in 2012 (12), Israel in 2015 (42), and Lebanon in 2018. UAE, Iran, and Turkey have taken some steps in this regard. (http://ipopi.org/).

## Peculiarities of Africa

Africa is a densely inhabited continent with an average inbreeding of around 35.4% (35) and surpassing 60% in parts of North and Sub-Saharan Africa (43). Although Africa is expected to have 988,000 PID cases, barely 2,500 patients have been diagnosed (36). The lack of resources, the absence of neonatal screening programs, and the need to enhance immunologic expertise are among the problems that hinder good medical care for PID. However, a steady rise in the number of reported PID cases is noticeable in between 2013 (1,463 patients) and 2018 (1,836 patients) with an increase rate of 25.6% (3). These advances are observed in countries where awareness programs are conducted such as Egypt, Tunisia, Algeria, Morocco, and Sudan (43).

## Status in Egypt

Egypt is considered one of the largest countries in the MENA region and has the largest population. The university hospitals carry the main brunt of identification of PID cases. In 2010, with the implementation of the WHO program of surveillance for vaccine-derived poliovirus in PID patients (iVDPV) (44), the Ministry of Health and Population has become increasingly involved in the surveillance for PID cases. Data on the prevalence of PIDs in Egypt is lacking as there is no national registry to date. The ESID database contains data from Egypt and some information can also be gained from the few published studies (33, 45–47). CID is considered the most common disease (31%), followed by predominantly antibody deficiency (30%), well-defined syndromes with PID (17.5%), and, finally, phagocytic defects (8.1%). The diagnostic lag mean was 29.9 months (45) in 2008 and improved to 1.67 years in 2016 (33).

Egypt still uses oral polio vaccine (OPV). In 2018, a dose of IPV (inactivated poliovirus vaccine) was introduced at the age of 4 months to the routine immunization schedule of infants. All newborns are given BCG and OPV at birth. This put, the as yet undiagnosed PID patient is at a significant risk of developing BCGosis and vaccine-associated paralytic poliomyelitis. In a study conducted on 130 patients with suspected or confirmed PID disorders, 6 patients were excreting VDPV, in which 5 of them had SCID and had X-linked agammaglobulinemia. Three patients developed acute flaccid paralysis (48). This underscores the importance of implementing the newborn screening programs, despite limited resources, for early diagnosis of PIDs and prevention of vaccination complications.

In a study on healthy Egyptian children, the level of TRECs and KRECs was found to be inversely proportional to age (46). The lowest threshold of TRECs (copies/μl blood) was 25 for the group aged 1 day to 5 months, and 24 for the group aged 5 months to 2.4 years. As for KRECs, the lowest threshold was 31 and 52, respectively (28).

## Discussion

PIDs are still underdiagnosed, and there is still a considerable diagnosis lag even in developed countries. Late diagnosis has major morbidity and mortality effects, and the establishment of screening programs for early detection and management becomes imperative.

In the absence of newborn screening programs for PIDs in many countries including Egypt, “clinical pattern recognition” (49) of various PIDs becomes a necessity, yet this would not supplant the need for neonatal screening programs. Many PIDs manifest in the neonatal period such as SCID, leucocyte adhesion deficiency (LAD), severe congenital neutropenia (SCN), chronic granulomatous disease (CGD), and defects in innate immunity. The diagnosis can be challenging at this age, and the difficulty, in part, stems from the natural immaturity of the neonatal immune system that may mask immune deficits and/or complicate interpretation of clinical findings and laboratory assays (50). The anatomic characteristics such as weak mucosal barriers, impaired Th1 cytokine production and immature cell-mediated immunity renders the newborn at risk of infection (50). The newborn has both qualitative and quantitative defects in complement (51). In sepsis, neutrophil count often falls due to exhaustion of the bone marrow reserves, which could be misdiagnosed as congenital neutropenia (51).

The most predictive factor for PID diagnosis is a family history of immunodeficiency, either confirmed or suspected, leading to early death or recurrent/chronic illness in one or more family members. Warning signs for PID in neonates have been suggested by the Jeffrey Modell Foundation (http://www.info4pi.org) and was modified by O'Connell (53). SCID patients could present with resistant or opportunistic infections. The presence of erythroderma should raise the suspicion of Omenn syndrome (54). The presence of eczema along with severe/recurrent infections in a neonate should raise the suspicion of certain diseases such as immunodeficiency polyendocrinopathy X-linked (IPEX), Wiskott-Aldrich syndrome (WAS), hyper-immunoglobulin E syndrome (HIES) (55), and DOCK 8 deficiency (56). Each of the previously described disease has an additional special character, such as microthrombocytopenia with WAS and staph infections with HIES. Mucosal abnormalities such as thrush and mouth ulcerations might mirror the underlying defect as STAT3 gain-of-function mutation. Delayed separation of the umbilical stump beyond 2 weeks is characteristic of LAD but may also be seen in other disorders such as IL-1 receptor kinase-4 (IRAK4) deficiency, SCN or CGD (51). Characteristic facial features along with cardiac defects with hypocalcemia suggest DiGeorge syndrome or CHARGE association (52). Thus, PID must be considered in the presence of certain syndromic features and abnormal facies. Presence of autoimmunity could be a clue for diseases such as IPEX. Enteritis in a neonate might be due to IL-10 and IL-10 receptor deficiencies (53).

A rare feature of PIDs is hydrops fetalis reported in IPEX (57) and X-linked lymphoproliferative disease type 2 (58). Hence, genetic testing is indicated in neonates with unexplained hydrops. Hepatosplenomegaly in the neonatal period has been described in hemophagocytic lymphohistiocytosis, and CGD (59). As confirmation of the importance of clinical identification of PID patients, the survival of patients diagnosed clinically was identical to those diagnosed by NBS, positive family history, or both (60).

Once a neonate is suspected of having a PID disorder, further investigations for the immune system should be done. Initial screening tests include complete blood counts. Quantitative Ig levels though are less informative in newborn and young infants because of reduced production and the transplacental transfer of IgGs. Lymphocyte subset enumeration by flow cytometry is indicated if SCID is suspected even in the setting of a normal lymphocyte count. The lymphoproliferative response to mitogens is the primary test for evaluating T cell function in newborns (61). In July 2019, the World Health Organization placed immunoglobulin quantitation and lymphocyte subset enumeration on the WHO list of essential *in vitro* diagnostics^2^.

Finally, newborn screening is essential for early diagnosis and management of PIDs, reducing morbidities, and perhaps saving lives of some PIDs especially SCIDs. However, still there are no available screening tests for many other PIDs, and their diagnosis requires clinical expertise. Although the importance of implementing screening programs cannot be outweighed, the lack of resources dictates that educational programs and disease-focused awareness campaigns be adopted. Appropriate support should be given for the provision of immunological and molecular diagnostic tools. In the meantime, dedicated technical efforts and funds and harmonization of international cooperation to achieve alignment with national health agendas will help overcome problems with newborn screening programs.

## Author Contributions

ZE-S conceived this review. ZE-S and NR participated in searching and collecting the sources, drafting, and final approval of the manuscript.

### Conflict of Interest

The authors declare that the research was conducted in the absence of any commercial or financial relationships that could be construed as a potential conflict of interest.

## Footnotes

^1^MENA countries include the following: Saudi Arabia, Kuwait, Bahrain, Qatar, United Arab Emirates, Oman, Yemen, Jordan, Iraq, Israel, Palestine, Syria, Lebanon, Iran, Turkey, Egypt, Sudan, Tunisia, Libya, Algeria, Morocco, and Mauritania.

^2^https://www.who.int/medical_devices/publications/Standalone_document_v8.pdf?ua=1 (accessed September 1, 2019).
